# siRNA‐induced CD44 knockdown suppresses the proliferation and invasion of colorectal cancer stem cells through inhibiting epithelial–mesenchymal transition

**DOI:** 10.1111/jcmm.17221

**Published:** 2022-03-01

**Authors:** Weiyan Zou, Yi Zhang, Guangfu Bai, Jialu Zhuang, Lin Wei, Zishu Wang, Meiqun Sun, Junbin Wang

**Affiliations:** ^1^ 74539 Department of Histology and Embryology Bengbu Medical College Bengbu City China; ^2^ The Second Department of Surgery Xiamen Hospital Affiliated to Beijing University of Chinese Medicine Xiamen City China; ^3^ Department of Emergency Wuxi Huishan District People’s Hospital Wuxi City China; ^4^ 74539 The Second School of Clinical Medicine Bengbu Medical College Bengbu City China; ^5^ Department of Oncology The First Affiliated Hospital of Bengbu Medical College Bengbu City China

**Keywords:** cancer stem cell, CD44, colorectal cancer, epithelial–mesenchymal transition, invasion, migration, proliferation

## Abstract

CD44 has shown prognostic values and promising therapeutic potential in multiple human cancers; however, the effects of CD44 silencing on biological behaviors of cancer stem cells (CSCs) have not been fully understood in colorectal cancer. To examine the contribution of siRNA‐induced knockdown of CD44 to the biological features of colorectal CSCs, colorectal CSCs HCT116‐CSCs were generated, and CD44 was knocked down in HCT116‐CSCs using siRNA. The proliferation, migration and invasion of HCT116‐CSCs were measured, and apoptosis and cell‐cycle analyses were performed. The sensitivity of HCT116‐CSCs to oxaliplatin was tested, and xenograft tumor growth assay was performed to examine the role of CD44 in HCT116‐CSCs tumorigenesis *in vivo*. In addition, the expression of epithelial–mesenchymal transition (EMT) markers E‐cadherin, N‐cadherin and vimentin was quantified. siRNA‐induced knockdown of CD44 was found to inhibit the proliferation, migration and invasion, induce apoptosis, promote cell‐cycle arrest at the G1/G0 phase and increase the sensitivity of HCT116‐CSCs to oxaliplatin in HCT116‐CSCs, and knockdown of CD44 suppressed *in vivo* tumorigenesis and intrapulmonary metastasis of HCT116‐CSCs. Moreover, silencing CD44 resulted in EMT inhibition. Our findings demonstrate that siRNA‐induced CD44 knockdown suppresses the proliferation, invasion and *in vivo* tumorigenesis and metastasis of colorectal CSCs by inhibiting EMT.

## 1 INTRODUCTION

Cancer stem cells (CSCs) are a small and elusive population of undifferentiated cancer cells that are characterized by self‐renewal capability, clonogenic growth and long‐term repopulation potential.^1^ This subpopulation of tumor cells has been identified in multiple types of cancers,^2^ including colorectal cancer,^3^ one of the most common malignancies with high morbidity and mortality worldwide.^4^

Previous studies have shown that CSCs play important roles in cancer initiation, growth, migration, invasion, metastasis and recurrence, and contribute to resistance to chemotherapy, radiotherapy and targeted therapy.^5^, ^6^, ^7^, ^8^, ^9^ Thus, elimination of CSCs may reverse the resistance to chemotherapy, radiotherapy and targeted therapy, improve the prognosis and yield long‐lasting responses in cancer patients.^10^, ^11^, ^12^ Colorectal CSCs have been therefore considered as a promising therapeutic target for colorectal cancer.^12^, ^13^, ^14^

Identification of CSCs is a prerequisite to the therapeutic use of these specific cells.^15^ Currently, the biomarkers for identification of CSCs mainly include CD molecules (CD133, CD166, CD44, CD24 and CD138), ATP‐binding cassette (ABC) transporters (ABCG2 and ABCB5), EpCAM, ALDH1 and CXCR4, Lgr5, ALDH1, Msi‐1, DCAMLK1 and EphB receptors, in which CD molecules are the most common markers for identifying CSCs.^16^, ^17^, ^18^

CD44, one of the most common CSC surface marker, is widely accepted as a key regulator of cancer stemness.^19^, ^20^ In addition, CD44 has shown prognostic values and promising therapeutic potential in multiple human cancers.^21^, ^22^, ^23^, ^24^ However, the effects of CD44 silencing on biological behaviors of CSCs remain to be investigated in colorectal cancer. This study was therefore designed with aims to examine the contribution of siRNA‐induced knockdown of CD44 to the biological features of colorectal CSCs.

## 2 MATERIALS AND METHODS

### 2.1 Animals

Four‐week‐old male athymic BALB/c nude mice were purchased from Nanjing Experimental Animal Center of the Chinese Academy of Sciences (Nanjing, China). All animals were maintained in a specific pathogen‐free facility and given free access to water and food.

### 2.2 Cell culture and HCT116‐CSCs preparation

Human colorectal cancer HCT116 cell line was purchased from the Cell Bank of Chinese Academy of Sciences (Shanghai, China) and cultured in McCoy's 5A medium (Invitrogen) supplemented with 10% fetal bovine serum (FBS; GIBCO), 100‐IU/ml penicillin (GIBCO) and 100‐μg/ml streptomycin (GIBCO). HCT116‐CSCs were enriched from HCT116 cells with the continuous cell microsphere culture and incubated in complete DMEM/F12 medium containing B27 (10 ng/ml), epidermal growth factor (EGF; 20 ng/ml), basic fibroblast growth factor (bFGF; 10 ng/ml) and leukemia inhibitory factor (LIF; 10 ng/ml). Briefly, log‐phase HCT116 cells were harvested and digested with pancreatin containing 0.25% EDTA and terminated with serum‐containing medium. Following centrifugation, the supernatant was discarded, and the sediment was washed twice with PBS, and re‐suspended in complete stem cell culture. The number of cells was counted. Cells were then seeded onto ultra‐low adhesive petri dishes at a density of 1 × 10^4^ cells/ml and incubated at 37°C in a humidified atmosphere containing 5% CO_2_. Semiquantitative medium changes were done once every 2–3 days, and cell passage was completed once the microsphere formation was observed to become larger and the structures to become loose. The culture medium containing microspheres in the petri dishes was collected during passaging, centrifuged at a low speed (500–700 r/min), and the supernatant was discarded. The microspheres were digested with a small amount of 0.25% trypsin‐EDTA (100–200 μl) according to the amount of cells, and the centrifuge tube was flicked. The microspheres were observed to be digested into a single‐cell suspension under an inverted microscope, and PBS was used to resuspend cells with 20 times of the amount of trypsin digestion, centrifuged at 1000 r/min for 5 min and washed twice with PBS. The number of cells was counted. Cells were then incubated in completely stem cell culture at a density of <10^4^ cells/ml. This method was used to subculture microsphere cells for at least 10 passages to enrich CSCs from HCT116 cells, which were named as HCT116‐CSCs.

### 2.3 Cell transfection

HCT116‐CSCs were incubated in completely stem cell culture without double antibodies. Cells were seeded onto 6‐well plates at a density of 1 × 10^5^ cells/well and incubated for 6 h. HCT116‐CSCs were transfected with three individual CD44 siRNAs (CD44‐siRNA 1#, 2# and 3#; Invitrogen) with the buffer reagent (RiboBio) according to the manufacturer's instructions, while a scrambled siRNA (si‐NC; Invitrogen) served as a negative control. At 48 h posttransfection, cells were harvested for the subsequent experiments.

### 2.4 MTT assay

The cell viability was measured using MTT assay every 24 h with the Cell Proliferation Reagent Kit I (Roche Applied Science) according to the manufacturer's protocol, and the half‐maximal inhibitory concentration (IC_50_) was calculated. Briefly, HCT116‐CSCs were seeded onto 96‐well plates (Corning, Inc.) at a density of 3000 cells/well and transfected with siRNAs. Cells were then seeded onto 96‐well plates at a density of 3 × 10^3^ cells/well, harvested in standard medium overnight, and treated with oxaliplatin (Jiangsu Hengrui Medicine Co., Ltd.) at concentrations of 0, 1, 5, 10, 15, 20, 30 and 40 μg/ml). To test the cell viability following oxaliplatin treatment, HCT116‐CSCs were plated in 96‐well plates at a density of 3000 cells/well and transfected with si‐NC and CD44‐siRNA 1# for 48 h. Transfected cells were then seeded onto 96‐well plates at a density of 3 × 10^3^ cells/well, harvested in standard medium overnight and treated with oxaliplatin at concentrations of 1.5 or 3 μg/ml (Jiangsu Hengrui Medicine Co., Ltd.), while untreated cell served as controls.

Following incubation for 48 h, cells were exposed to MTT solutions (0.5 mg/ml; Sigma‐Aldrich) for further 4 h, and then, the medium was substituted with 150‐μl dimethyl sulfoxide (DMSO; Sigma‐Aldrich) and vortexed for 10 min. The absorbance of each well was measured at 490 nm. In addition, the cell viability was evaluated at 0, 24, 48, 72 and 96 h using 0.5‐mg/ml MTT solution without oxaliplatin treatment. Each assay was repeated at least in triplicate.

### 2.5 Colony formation assay and migration and invasion assays

For the colony formation assay, a total of 600 transfected cells were seeded onto 6‐well plates (Corning, Inc.) and maintained in DMEM‐F12 medium supplemented with 10% FBS for 2 weeks, displacing the medium every 3–4 days. After incubation for 14 days, cells were fixed with methanol and stained with 0.1% crystal violet (Sigma‐Aldrich). Visible colonies were then counted. Triplicate wells were assessed for each treatment group, and experiments were independently repeated in triplicate.

For cell migration and invasion assays, we used 24‐well Transwell chambers with 8‐μm pore size polycarbonate (Millipore) to test cell migratory ability. Briefly, 8 × 10^4^ transfected cells in serum‐free DMEM‐F12 medium were transferred into the upper chamber of an insert with Matrigel (1:8 ratio) or not, and DMEM‐F12 medium supplemented 10% FBS was added to the lower chamber. After incubation for 36 h, the cells remaining on the upper membrane were removed with a cotton wool, and the cells that had migrated or invaded through the other side of the membrane surface were fixed with methanol and stained with 0.1% crystal violet (Sigma‐Aldrich). Five random fields were imaged and counted under an inverted microscope (Olympus). The assays were independently repeated in triplicate.

### 2.6 Flow cytometry

HCT116‐CSCs of P2 generation were seeded onto 6‐well plates at a density of 1 × 10^5^ cells/well and incubated with complete stem cell culture for 48 h after transfection with siRNAs or si‐NC by trypsinization. After double staining with FITC‐Annexin V and propidium iodide (PI) according to the manufacturer's recommendations, cells were analysed with a FACScan^®^ flow cytometer (BD Biosciences) equipped with CellQuest software (BD Biosciences). Cells were classified into viable cells, dead cells, early apoptotic cells and apoptotic cells, and the relative ratio of early apoptotic cells was compared with control transfection from each experiment. For cell‐cycle analysis, cells were stained with PI using the CycleTEST PLUS DNA Reagent Kit (BD Biosciences) following the manufacturer's instructions and then analysed with a FACScan flow cytometer. The percentage of cells at G0/G1, S and G2/M phases were counted and compared.

### 2.7 qPCR assay

Total RNA was extracted from cells or tissues with the TRIzol reagent (Invitrogen) following the manufacturer's instructions. Total RNA (1 μg) was reversely transcribed into cDNA using the PrimeScript RT reagent kit (TaKaRa), and the *CD44*, *E*‐*cadherin*, *N*‐*cadherin* and *vimentin mRNA* expression was quantified with the SYBR Premix Ex Taq (TaKaRa) using the designed primers (Table 1) on an ABI 7500 real‐time PCR system (Applied Biosystems), while glyceraldehyde‐3‐phosphate dehydrogenase (*GAPDH*) served as an internal control. The relative gene expression was estimated using the 2^−ΔΔCT^ method. All assays were performed in triplicate.

**TABLE 1:** Primers used for qPCR assay

Gene	Sequences
*CD44*	Forward: 5′‐TATAACCTGCCGCTTTGCGA−3′; Reverse: 5’‐CAGGTCTCAAATCCGATGC −3’.
*E‐cadherin*	Forward: 5′‐GAACGCATTGCCACATACAC−3′; Reverse: 5′‐GAGGATGGTGTAAGCGATGG−3′.
*Vimentin*	Forward: 5′‐GTACCGGAGACAGGTGCAGT−3′; Reverse: 5′‐CTCAATGACAAGGGCCATCT−3′.
*N‐cadherin*	Forward: 5′‐ATGGAAGGCAATCCCACATA−3′; Reverse: 5′‐CAGTAGGATCTCCGCCACTG−3′.
*GAPDH*	Forward: 5′‐AGCCACATCGCTCAGACAC−3′; Reverse: 5′‐GCCCAATACGACCAAATCC−3′.

### 2.8 Western blotting assay

Transfected HCT116‐CSCs were harvested and lysed with RIPA extraction reagent (Solarbio) supplemented with a protease inhibitor cocktail (Solarbio) and phenylmethylsulfonyl fluoride (Solarbio). Equal amount of total protein (30 μg) was separated by 10% SDS‐polyacrylamide gel electrophoresis (SDS‐PAGE), transferred to the PVDF membranes with 0.22 μm in pore size (Millipore) and then incubated with specific antibodies against E‐cadherin (1:1000; GeneTex), N‐cadherin (1:1000; GeneTex) and vimentin (1:1000; GeneTex) at 4°C overnight, while the anti‐GAPDH antibody (1:5,000; CMCTAG, Inc.) served as a loading control. The immunoblots were then incubated with secondary antibodies. ECL chemiluminescence substrate (Millipore) was used for quantification by densitometry with the Quantity One software (Bio‐Rad Laboratories, Inc.).

### 2.9 Xenograft tumor growth assay

To examine the impact of CD44 knockdown on *in vivo* tumorigenesis and metastasis of HCT116‐CSCs, HCT‐CSCs were stably transfected with shRNA‐CD44 and sh‐NC (Dharmacon, Inc.), and digested with pancreatin, seeded onto petri dishes and incubated with 2‐ to 10‐μg/ml puromycin (Sigma‐Aldrich). Medium containing fresh puromycin was changed once every 3–4 days, and cells growing to approximately 90% confluence were passaged for 1–2 weeks. HCT116‐CSCs stably transfected with shRNA‐CD44 and sh‐NC were then harvested at a concentration of 8 × 10^5^ cells/ml and subcutaneously injected into the back of the axilla of each mouse (100 μl per mouse). Tumorigenesis was observed daily in each mouse, and the volume of xenograft tumors was measured if xenograft tumors were visible, followed by once measurement every 5 days. The tumor volume was calculated using the following formula: *V* = *W* ^2^ × *L*/2, where *V* indicates the tumor volume, *W* means the shortest diameter of the xenograft tumor and *L* means the longest diameter. Mice were sacrificed 6 weeks postinjection, and the xenograft tumor weight was measured. The primary tumors were excised, and tumor tissues were used for HE staining and immunostaining analysis. Lung specimens were excised from each mouse, and intrapulmonary metastatic nodules were observed under a microscope.

### 2.10 Immunostaining analysis

Immunostaining analysis was performed for the detection of Ki‐67, E‐cadherin, N‐cadherin and vimentin expression as described previously.^25^ Positive E‐cadherin and N‐cadherin expression was defined as the presence of brown granules in the tumor cell membrane and cytoplasm, and positive vimentin expression was considered in the presence of brown granules in the tumor cell cytoplasm, while positive Ki‐67 expression was considered if tan nuclear staining was seen.

### 2.11 Data management

All measurement data were described as mean ±standard error (SE). Data were tested for statistical significance with the Student's *t* test, one‐way analysis of variance (ANOVA) and the Mann‐Whitney *U* test. All statistical analyses were performed using the statistical software SPSS version 21.0 (SPSS, Inc.), All data analyses were done using the software GraphPad Prism version 5.0, with a *p* value of <0.05 indicative of statistical significance.

### 2.12 Ethics approval

The study protocol was reviewed and approved by the Ethics Review Committee on Animal Experiments of Bengbu Medical College (approval no. 2020‐177). All animal procedures described in this study were performed strictly following the Guide for the Care and Use of Laboratory Animals and Chinese Animal Management Regulations (2017 revised version). All efforts were made to reduce the number of laboratory animals used in this study and minimize animal suffering during the experimental procedures.

## 3 RESULTS

### 3.1 Knockdown efficiency of CD44 expression in HCT116‐CSCs

**FIGURE 1:**
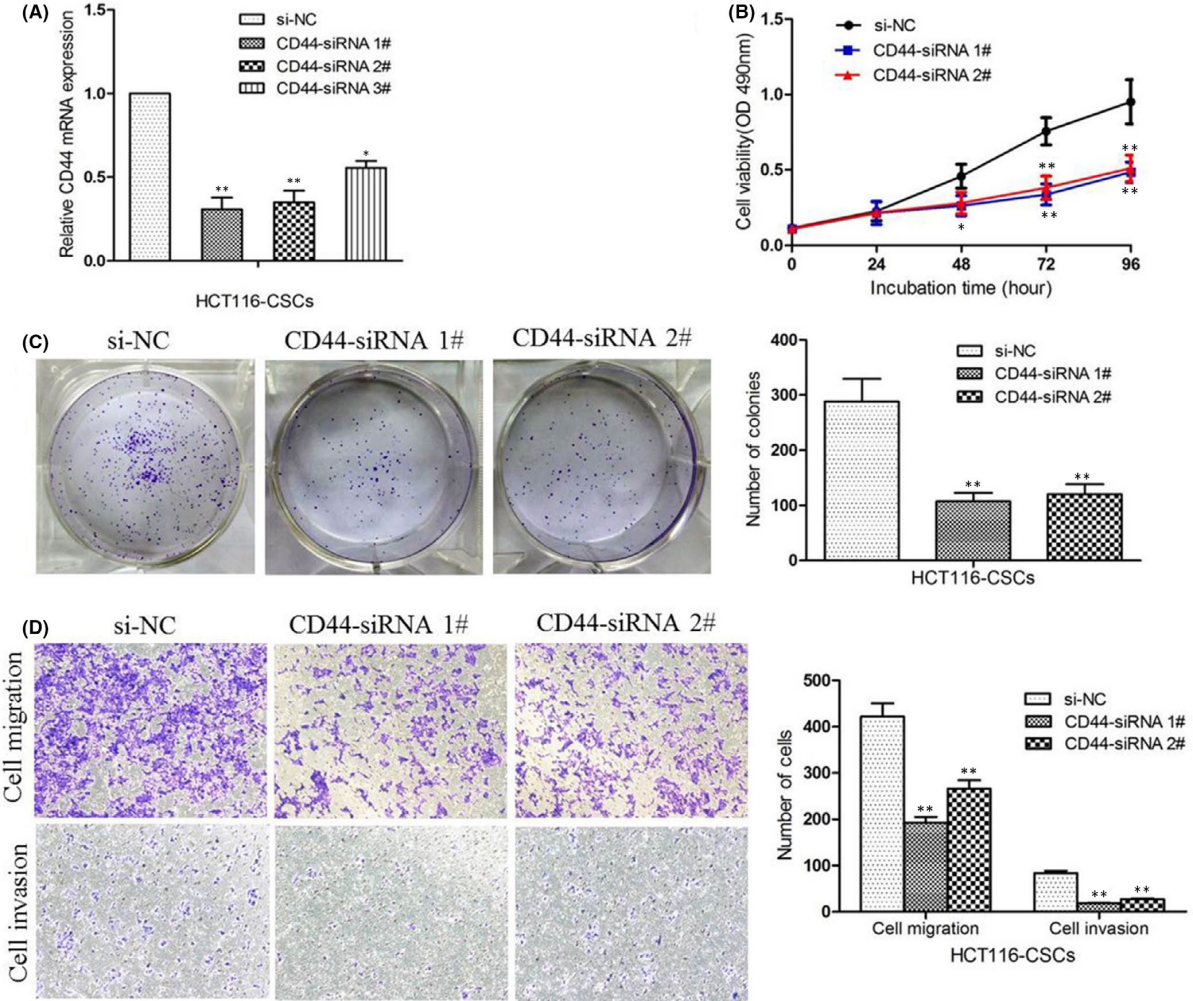
CD44 silencing inhibited HCT116‐CSCs proliferation, migration and invasion. (A) qPCR assay quantifies the knockdown efficiency of CD44 expression in HCT116‐CSCs; (B) MTT assay reveals that the proliferation of HCT116‐CSCs is significantly inhibited following CD44‐siRNA transfection relative to si‐NC transfection; (C) colony formation assays show that knockdown of CD44 expression results in a reduction in clonogenic survival of HCT116‐CSCs; (D) Transwell migration and invasion assays reveal that knockdown of CD44 expression inhibits the migration and invasion of HCT116‐CSCs compared with si‐NC transfection. *p < 0.05; **p < 0.01

To investigate the knockdown efficiency of CD44 expression in HCT116‐CSCs, qPCR assay was performed to detect CD44 expression in HCT116‐CSCs transfected with different CD44 siRNAs 48 h posttransfection. A greater knockdown efficiency was seen by CD44‐siRNA 1# and 2# than by CD44‐siRNA 3# (Figure 1A), and CD44‐siRNA 1# and 2# were therefore selected for the subsequent experiments.

### 3.2 CD44 silencing inhibited HCT116‐CSCs proliferation, migration and invasion

To evaluate the functional role of CD44 in HCT116‐CSCs, we first examined the effect of CD44 silencing on cell proliferation. MTT assays revealed that cell proliferation was significantly inhibited in HCT116‐CSCs following CD44‐siRNA transfection (Figure 1B). Colony formation assays then showed that knockdown of CD44 expression suppressed the colony formation of HCT116‐CSCs, which reflected the self‐renewal and differentiation abilities of the CSCs (Figure 1C). Furthermore, Transwell migration and invasion assays revealed that siRNA‐induced knockdown of CD44 expression inhibited the migration and invasion of HCT116‐CSCs as compared to si‐NC (Figure 1D). These data demonstrate that CD44 knockdown inhibits the migratory phenotype of HCT116‐CSCs.

### 3.3 Knockdown of CD44 induces apoptosis and promotes cell‐cycle arrest at the G1/G0 phase of HCT116‐CSCs

**FIGURE 2:**
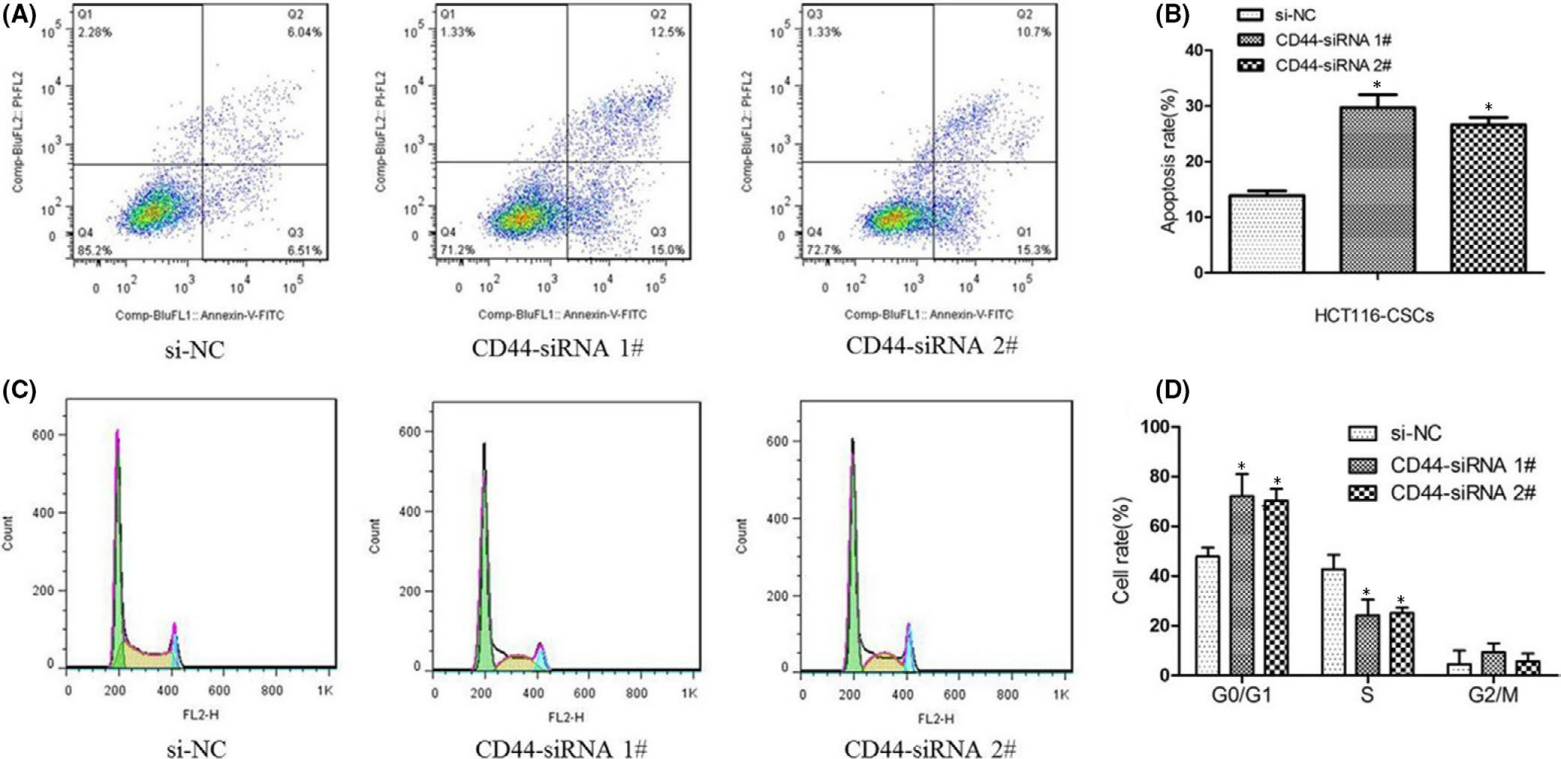
Knockdown of CD44 induces apoptosis and promotes cell‐cycle arrest at the G1/G0 phase of HCT116‐CSCs. (A) flow cytometric analysis detects apoptosis of HCT116‐CSCs transfected with CD44‐siRNA and si‐NC; (B) greater apoptotic rates of HCT116‐CSCs transfected with CD44‐siRNA are detected as compared to si‐NC; (C) flow cytometric analysis detects the number of HCT116‐CSCs transfected with CD44‐siRNA and si‐NC at G0/G1, S and G2/M phases; (D) flow cytometry detects higher proportions of HCT116‐CSCs transfected with CD44‐siRNA relative to si‐NC at the G0/G1 phase and lower proportions of HCT116‐CSCs transfected with CD44‐siRNA relative to si‐NC at the S phase. *p < 0.05

To further detect the effects of CD44 knockdown on apoptosis and cell cycle of HCT116‐CSCs, flow cytometric analysis was performed. Flow cytometry detected that CD44 knockdown resulted significantly higher apoptotic rates of HCT116‐CSCs than si‐NC (Figure 2A,B). In addition, silencing of CD44 expression was found to induce the cell‐cycle arrest at the G1/G0 phase in HCT116‐CSCs and cause a reduction in the number of HCT116‐CSCs in the S phase (Figure 2C,D). Taken together, our data demonstrate that knockdown of CD44 induces apoptosis and promotes cell‐cycle arrest at the G1/G0 phase in HCT116‐CSCs.

### 3.4 Knockdown of CD44 promotes the sensitivity of HCT116‐CSCs to oxaliplatin

**FIGURE 3:**
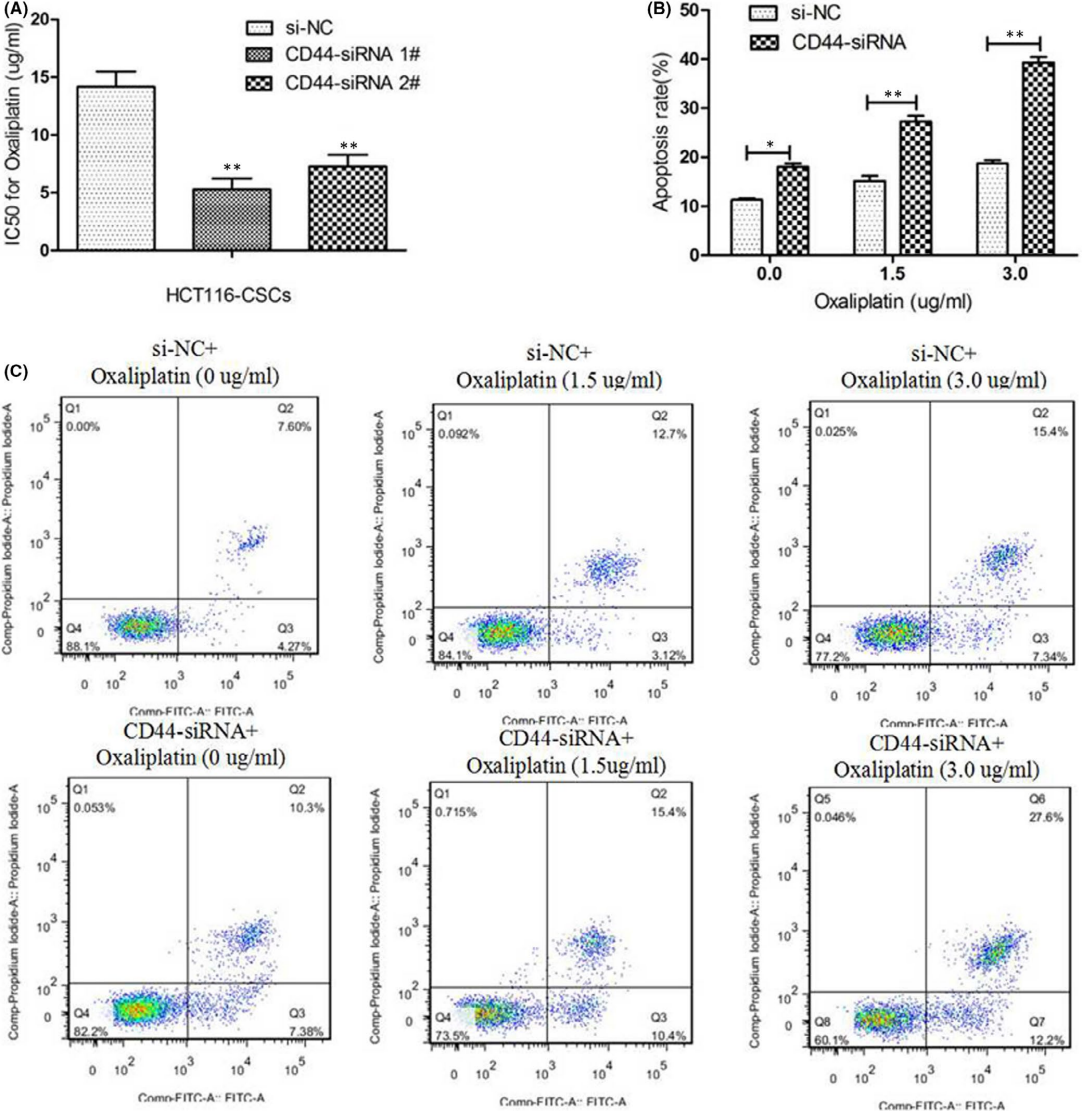
Knockdown of CD44 promotes the sensitivity of HCT116‐CSCs to oxaliplatin. (A) MTT assay measures lower oxaliplatin IC_50_ values (5.28 ± 1.64 and 7.25 ± 1.81 μg/ml) against HCT116‐CSCs transfected with CD44‐siRNA relative to si‐NC (14.15 ± 2.31 μg/ml) against HCT116‐CSCs; (B) higher apoptotic rates are detected in HCT116‐CSCs transfected with CD44‐siRNA 1# than those transfected with si‐NC following exposure to oxaliplatin at concentrations of 0, 1.5 and 3.0 μg/ml; (C) flow cytometric analysis of HCT116‐CSCs transfected with CD44‐siRNA 1# and si‐NC following treatment with oxaliplatin at concentrations of 0, 1.5 and 3.0 μg/ml. *p < 0.05; **p < 0.01

We then examine the effect of CD44 knockdown on the sensitivity to oxaliplatin in HCT116‐CSCs. MTT assay measured that siRNA‐induced CD44 knockdown reduced the oxaliplatin IC_50_ values (5.28 ± 1.64 and 7.25 ± 1.81 μg/ml) as compared to si‐NC (14.15 ± 2.31 μg/ml) against HCT116‐CSCs (Figure 3A). In addition, a more significant reduction was seen in the viability of HCT116‐CSCs transfected with CD44‐siRNA 1# with the increase of oxaliplatin doses (Figure S1).

Following exposure to oxaliplatin at concentrations of 0, 1.5 and 3.0 μg/ml in HCT116‐CSCs transfected with si‐NC or CD44‐siRNA 1#, flow cytometry detected higher apoptotic rates of HCT116‐CSCs transfected with CD44‐siRNA 1# than those transfected with si‐NC regardless of oxaliplatin treatment at different concentrations (Figure 3B,C). These data indicate that CD44 silencing increases the sensitivity to oxaliplatin in HCT116‐CSCs through inducing cell apoptosis.

### 3.5 Knockdown of CD44 inhibits epithelial–mesenchymal transition (EMT) in HCT116‐CSCs

**FIGURE 4:**
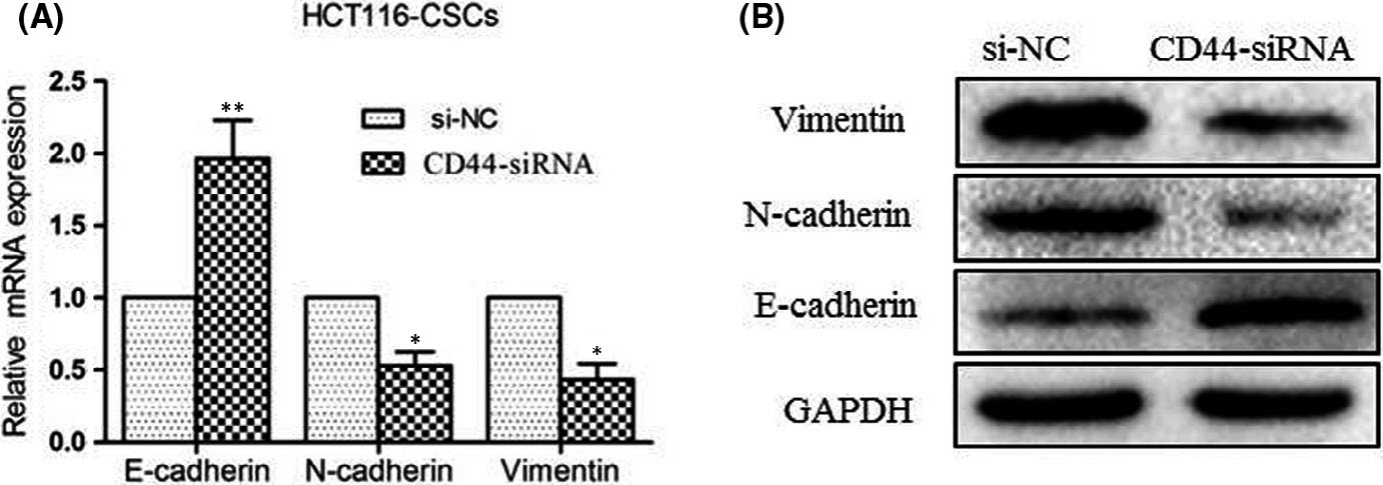
Knockdown of CD44 inhibits epithelial–mesenchymal transition (EMT) in HCT116‐CSCs. (A) qPCR detects lower relative expression of *N*‐*cadherin* and *vimentin* and higher relative *E*‐*cadherin* expression in HCT116‐CSCs following CD44 knockdown; (B) Western blotting determines lower N‐cadherin and vimentin expression and higher E‐cadherin expression in HCT116‐CSCs following CD44 knockdown. *p < 0.05; **p < 0.01

As described above, Transwell migration and invasion assays showed that the metastatic ability of HCT116‐CSCs was significantly weakened by CD44 silencing (Figure 1D). Next, we examined the effects of CD44 knockdown on EMT through detecting the expression of EMT markers E‐cadherin, N‐cadherin and vimentin. qPCR detected that CD44 knockdown resulted in a reduction in the relative expression of *N*‐*cadherin* and *vimentin* and an increase in the relative *E*‐*cadherin* expression in HCT116‐CSCs (Figure 4A), and similarly, Western blotting determined lower N‐cadherin and vimentin expression and higher E‐cadherin expression in HCT116‐CSCs following CD44 knockdown (Figure 4B), indicating the inhibition of EMT. Our findings demonstrate that CD44 knockdown may suppress HCT116‐CSCs migration and invasion through inhibiting EMT.

### 3.6 Knockdown of CD44 inhibits HCT116‐CSCs tumorigenesis *in vivo*

**FIGURE 5:**
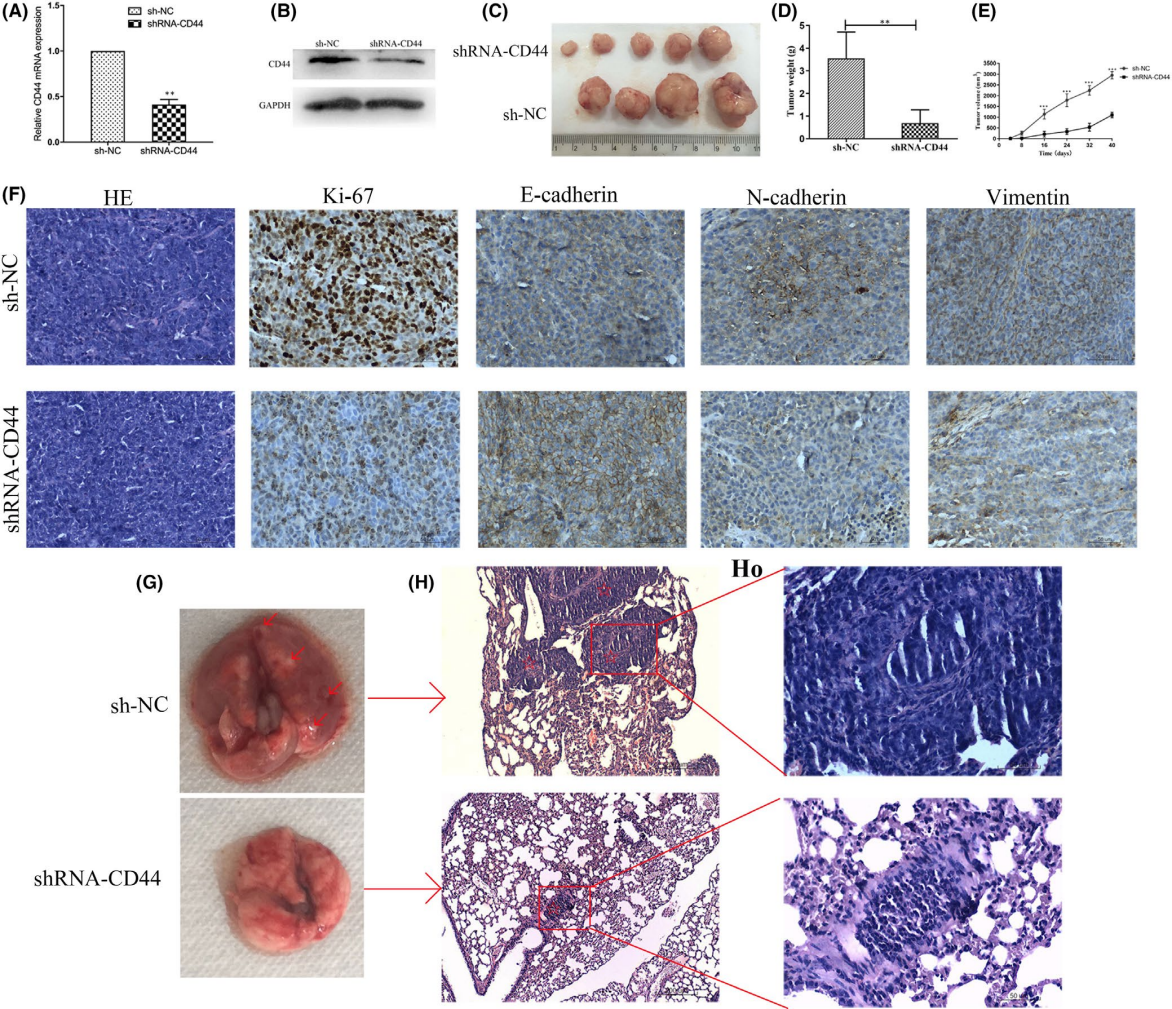
Knockdown of CD44 inhibits *in vivo* tumorigenesis and metastasis of HCT116‐CSCs. (A) qPCR assay quantifies significantly lower CD44 expression in shRNA‐CD44‐transfected HCT116‐CSCs than in sh‐NC‐transfected HCT116‐CSCs; (B) Western blotting determines lower CD44 expression in shRNA‐CD44‐transfected HCT116‐CSCs than in sh‐NC‐transfected HCT116‐CSCs; (C) presence of xenograft tumors at the injection site 42 days postinjection; (D) the mean weight of the xenograft tumors derived from shRNA‐CD44‐transfected HCT116‐CSCs is significantly lower than from sh‐NC‐transfected HCT116‐CSCs; (E) the mean volume of the xenograft tumors derived from shRNA‐CD44‐transfected HCT116‐CSCs are all significantly smaller than from sh‐NC‐transfected HCT116‐CSCs 8, 16, 24, 32 and 40 days postinfection; (F) immunostaining analysis detects lower Ki‐67, N‐cadherin and vimentin expression and higher E‐cadherin expression in xenograft tumors derived from shRNA‐CD44‐transfected HCT116‐CSCs than from sh‐NC‐transfected HCT116‐CSCs; (G) representative images of nude mouse lung 6 weeks following injection of HCT116‐CSCs. Intrapulmonary metastatic nodules are seen in mice subcutaneously injected with sh‐NC‐transfected HCT116‐CSCs, while apparent intrapulmonary metastatic nodules are not visible in mice subcutaneously injected with shRNA‐CD44‐transfected HCT116‐CSCs. The red arrows indicate the intrapulmonary metastatic nodules; (H) HE staining displays multiple intrapulmonary metastatic nodules in mice subcutaneously injected with shRNA‐CD44‐transfected HCT116‐CSCs, while intrapulmonary metastatic nodules are visible in only one nude mouse subcutaneously injected with sh‐NC‐transfected HCT116‐CSCs. The red pentagrams indicate intrapulmonary metastatic nodules. Magnification, 100 ×; H_O_, Figure 5H at a magnification of 400×. **p < 0.01; ***p < 0.001

Since CD44 knockdown was found to suppress HCT116‐CSCs migration and invasion *in vitro*, xenograft tumor growth assay was performed to examine the effects of CD44 knockdown on *in vivo* tumorigenicity and metastasis in nude mice. qPCR and Western blotting assays showed lower CD44 expression in HCT116‐CSCs transfected with shRNA‐CD44 than in those transfected with sh‐NC at both transcriptional and translational levels, confirming a high knockdown efficiency (Figure 5A,B). All mice were found to develop xenograft tumors at the injection site 42 days postinjection (Figure 5C), and the mean weight of the xenograft tumors derived from shRNA‐CD44‐transfected HCT116‐CSCs was significantly lower than from sh‐NC‐transfected HCT116‐CSCs (Figure 5D). During the 42‐day study period, the mean volumes of the xenograft tumors derived from shRNA‐CD44‐transfected HCT116‐CSCs were all significantly smaller than from sh‐NC‐transfected HCT116‐CSCs (Figure 5E). In addition, immunostaining detected lower Ki‐67, N‐cadherin and vimentin expression and higher E‐cadherin expression in xenograft tumors derived from shRNA‐CD44‐transfected HCT116‐CSCs than from sh‐NC‐transfected HCT116‐CSCs (Figure 5F).

Next, we observed intrapulmonary metastatic nodules in mice following subcutaneous injection of HCT116‐CSCs. Except one natural death in the sh‐NC group during the study period, intrapulmonary metastatic nodules were seen in other four mice, while one of the five mice presented intrapulmonary metastatic nodules in the shRNA‐CD44 group (Figure 5G,H,H_O_). Collectively, these findings demonstrate that CD44 knockdown suppresses *in vivo* tumorigenesis and metastasis of HCT116‐CSCs through inhibiting EMT.

## 4 DISCUSSION

CD44, a cell adhesion molecule, has shown an important role in tumor progression and metastasis.^26^ Previous studies have shown the roles of CD44 as prognostic factors and therapeutic targets in human cancers.^20^ Roosta and colleagues^27^ identified the clinical association of CD44 with the stage of breast cancer and systematic reviews and meta‐analyses revealed that CD44 expression is a prognostic factor for pharyngolaryngeal cancer,^28^ non‐small‐cell lung cancer,^29^ renal cell carcinoma^30^ and colorectal cancer.^31^ In addition, CD44 has been identified as a potential therapeutic target in head and neck squamous cell carcinoma,^32^ leukemia^33^ and other cancers.^34^

Targeting CSCs has been recognized as an emerging option for cancer therapy.^35^, ^36^, ^37^, ^38^ CD44, one of the most common CSC surface marker, is involved in the regulation of cancer cell stemness.^39^ The shortest CD44 isoform (CD44s) was found to inhibit breast cancer stemness, and the cleaved product of CD44 (CD44ICD) promoted breast cancer stemness,^40^ while nuclear CD44 in liver cancer stem cells is responsible for the poorly differentiated highly malignant tumor cells by maintenance of low stemness state.^41^ In addition, targeting colorectal CSCs is proposed to become a promising approach for the future cure of colorectal cancer.^42^ However, the impact of CD44 knockdown on the biological behaviors of CSCs has not been fully understood in colorectal cancer.

This study, designed in both *in vitro* and *in vivo* assays, aimed to investigate the effects of siRNA‐induced CD44 knockdown on the proliferation, migration, invasion, apoptosis and cell cycle of HCT116‐CSCs. CD44 silencing was found to suppress the proliferation, migration and invasion, induce apoptosis and promote cell‐cycle arrest at the G1/G0 phase in HCT116‐CSCs, which is similar to the findings seen in prostate cancer^43^ and pancreatic cancer.^44^ However, a recent study reported that CD44 knockdown promoted the proliferation and migration of claudin‐low MDA‐MB‐231 and Hs 578T breast cancer cell lines.^45^ This difference may be attributed to the various types of cell lines. We then measured the sensitivity of HCT116‐CSCs to oxaliplatin following CD44 silencing, a third‐generation platinum drug as first‐line chemotherapy for colorectal cancer.^46^ siRNA‐induced knockdown of CD44 was found to promote the sensitivity to oxaliplatin in HCT116‐CSCs, which is consistent with previous studies reporting that CD44 knockdown improved cisplatin sensitivity in non‐small‐cell lung cancer.^47^, ^48^ In addition, xenograft tumor growth assay revealed that CD44 knockdown inhibited *in vivo* tumorigenesis and metastasis of HCT116‐CSCs, which is in agreement with the reports seen in breast cancer^49^, ^50^ and head and neck squamous cell carcinoma.^51^

Epithelial–mesenchymal transition, which is characterized by reduced E‐cadherin and increased N‐cadherin and vimentin expression, has shown a critical role in cancer invasion and metastasis.^52^ γ‐glutamylcyclotransferase (GGCT) was reported to promote colorectal cancer migration and invasion via EMT,^53^ miR‐300 was found to promote colorectal cancer proliferation, migration and invasion via EMT,^54^ and schisandrin B attenuated cancer invasion and metastasis by inhibiting EMT.^55^ In this study, qPCR and Western blotting assay detected lower N‐cadherin and vimentin expression and higher E‐cadherin expression in HCT116‐CSCs at both translational and transcriptional levels following CD44 knockdown, and immunostaining analysis revealed lower N‐cadherin and vimentin expression and higher E‐cadherin expression in xenograft tumors derived from CD44‐siRNA‐transfected HCT116‐CSCs than in those from si‐NC‐transfected HCT116‐CSCs, indicating that CD44 knockdown inhibits EMT. Collectively, our data demonstrate that CD44 knockdown may suppress the proliferation, migration and invasion, induce apoptosis and promote cell‐cycle arrest at the G1/G0 phase and increase the sensitivity to oxaliplatin in HCT116‐CSCs through suppressing EMT.

In summary, the results of the present study demonstrate that siRNA‐induced CD44 knockdown suppresses the proliferation, migration and invasion of colorectal CSCs by inhibiting EMT. Our findings confirm that targeting colorectal CSCs is a promising therapy for colorectal cancer, and CD44 may be a novel therapeutic target for the treatment of colorectal cancer.

## CONFLICT OF INTEREST

The authors declare no conflict of interests.

## AUTHOR CONTRIBUTIONS

**Weiyan Zou:** Investigation (equal); Writing – original draft (equal). **Yi Zhang:** Investigation (equal). **Guangfu Bai:** Investigation (equal). **Jialu Zhuang:** Formal analysis (equal); Investigation (equal); Software (equal). **Lin Wei:** Formal analysis (equal); Investigation (equal); Software (equal). **Zishu Wang:** Investigation (equal). **Meiqun Sun:** Investigation (equal). **Junbin Wang:** Conceptualization (equal); Funding acquisition (equal); Supervision (equal); Validation (equal); Writing – review & editing (equal).

## Supporting information

Fig S1
