# Supplementary material for: siRNA‐induced CD44 knockdown suppresses the proliferation and invasion of colorectal cancer stem cells through inhibiting epithelial–mesenchymal transition
Source: J Cell Mol Med. 2022 Mar 1;26(7):1969–78. doi: 10.1111/jcmm.17221 (PMC8980945; doi:10.1111/jcmm.17221)
Supplement: Supplementary file 1 — Fig S1 [file JCMM-26-1969-s001.docx]

**Supplementary file.**

**Figure 1. Effects of oxaliplatin treatment on the viability of HCT116-CSCs.** To test the cell viability following oxaliplatin treatment, HCT116-CSCs were plated in 96-well plates at a density of 3,000 cells/well, and transfected with si-NC and CD44-siRNA 1# for 48 hours. Then, transfected cells were seeded onto 96-well plates at a density of 3 × 10^3^ cells/well, harvested in standard medium overnight, and treated with oxaliplatin at concentrations of 1.5 or 3 μg/ml (Jiangsu Hengrui Medicine Co., Ltd.; Lianyungang, China), while untreated cell served as controls. Five repeated wells are assigned for each dose, and the mean OD is estimated after the highest and lowest values are removed. MTT assay reveals a more significant reduction in the viability of HCT116-CSCs transfected with CD44-siRNA 1# with the increase of oxaliplatin dose relative of HCT116-CSCs transfected with si-NC.


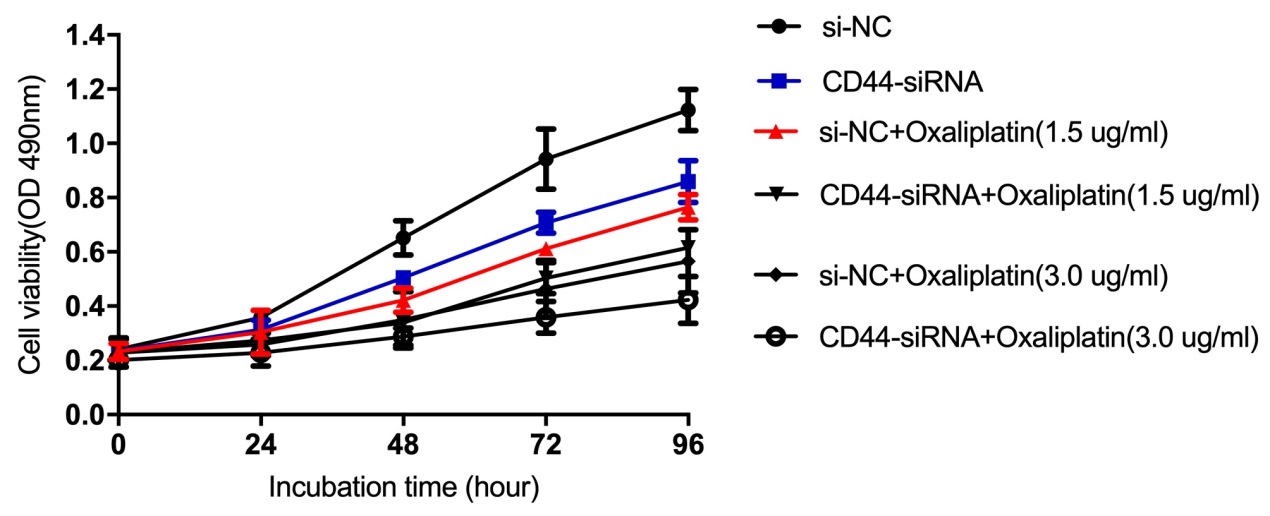


**Figure 1**
